# On the robustness of inference of association with the gut microbiota in stool, rectal swab and mucosal tissue samples

**DOI:** 10.1038/s41598-021-94205-5

**Published:** 2021-07-21

**Authors:** Shan Sun, Xiangzhu Zhu, Xiang Huang, Harvey J. Murff, Reid M. Ness, Douglas L. Seidner, Alicia A. Sorgen, Ivory C. Blakley, Chang Yu, Qi Dai, M. Andrea Azcarate-Peril, Martha J. Shrubsole, Anthony A. Fodor

**Affiliations:** 1 Department of Bioinformatics and Genomics, University of North Carolina at Charlotte, Charlotte, NC USA; 2 Department of Medicine, Vanderbilt University Medical Center, Nashville, TN USA; 3 Digestive Disease and Surgical Institute, Cleveland Clinic, Cleveland, OH USA; 4 Department of Biostatistics, Vanderbilt University Medical Center, Nashville, TN USA; 5 Department of Medicine and Microbiome Core Facility, School of Medicine, University of North Carolina, Chapel Hill, NC USA; 6 Department of Bioinformatics and Genomics, University of North Carolina at Charlotte, 9331 Robert D. Snyder Rd, Room 361, Charlotte, NC 28223 USA

**Keywords:** Metagenomics, Microbiome

## Abstract

The gut microbiota plays an important role in human health and disease. Stool, rectal swab and rectal mucosal tissue samples have been used in individual studies to survey the microbial community but the consequences of using these different sample types are not completely understood. In this study, we report differences in stool, rectal swab and rectal mucosal tissue microbial communities with shotgun metagenome sequencing of 1397 stool, swab and mucosal tissue samples from 240 participants. The taxonomic composition of stool and swab samples was distinct, but less different to each other than mucosal tissue samples. Functional profile differences between stool and swab samples are smaller, but mucosal tissue samples remained distinct from the other two types. When the taxonomic and functional profiles were used for inference in association with host phenotypes of age, sex, body mass index (BMI), antibiotics or non-steroidal anti-inflammatory drugs (NSAIDs) use, hypothesis testing using either stool or rectal swab gave broadly significantly correlated results, but inference performed on mucosal tissue samples gave results that were generally less consistent with either stool or swab. Our study represents an important resource for determination of how inference can change for taxa and pathways depending on the choice of where to sample within the human gut.

## Introduction

A growing number of studies have reported the essential roles of the human gut microbiota in human health and that microbiota alterations are associated with diseases including colorectal cancer, inflammatory bowel disease, obesity and diabetes^1–4^. The human colorectum is a complex system consisting of many microhabitats; studies have reported that the luminal and mucosal microbiota harbor heterogeneous microbial communities^5^. With the oxygen decline from the intestinal mucosa towards the lumen, anaerobic microorganisms are more abundant in luminal than mucosal environments^6–8^. On the other hand, the mucosal microbiota, directly adherent to the host tissue, may be more sensitive and respond more rapidly to localized changes in host tissues, compared to the luminal microbiota that is isolated from the loose mucus layer on the surface of the colorectal wall^9^.

Stool samples are the most common biospecimen used to assess composition and functionality of the human gut microbiota in human research because of the large amount of biomass and the feasibility of collection; however, stool-derived profiles are more representative of luminal microorganisms than of mucosa-associated microbes. Mucosal tissue biopsies better characterize mucosa-associated microbes but are less frequently used because of the invasive nature and accompanying risk of the procedure. Rectal swabs may be used when stool samples are not practical to obtain, for example in the intensive care unit, and may collect a combination of both luminal and mucosal communities^10^. While stool and mucosal samples are generally distinct, there are mixed findings on the similarity between stool and swab samples^11–13^. Thus, different biospecimen types may be needed to sample microorganisms residing in different niches or to reflect different physiological conditions. For example, a study on colitis-induced inflammation in mouse reported that microbial dysbiosis in the mucus layer was detected preceding colitis while changes in stool microbiota were detected post-colitis^9^.

Most of the studies assessing the variation of microbiota profile by biospecimen type have focused on taxonomic composition characterized by 16S rRNA gene amplicon sequencing. Previous literature of observed variation using shotgun metagenomics is usually limited by the sample size^10,14,15^. Compared to the 16S rRNA gene amplicon sequencing, shotgun metagenome sequencing utilizes total DNA instead of PCR products thus reducing the bias introduced during PCR amplification step^16,17^. Moreover, metagenome sequencing not only determines the taxonomic composition of the gut bacterial communities but also generates information on functional profiles. With the increasing application of shotgun metagenome sequencing in microbiota studies, a better understanding of the metagenome variation across biospecimen types will help investigators develop and interpret their experimental design.

In this study, we collected matched stool, rectal swab and rectal mucosal tissue samples from 240 study participants at up to two time points, which resulted in 1,397 shotgun metagenomes. This is one of the largest studies comparing metagenomes of human stool, rectal swab and rectal mucosal tissue samples. We estimated the biospecimen type variation of both metagenome taxonomy and functional pathways. We also assessed whether the associations between taxa/pathways and age, sex, body mass index (BMI), non-steroidal anti-inflammatory drugs (NSAIDs) use and antibiotics use were consistent across the different sample types.

## Results

### Taxonomic composition of metagenomes was associated with sample types

**Figure 1:**
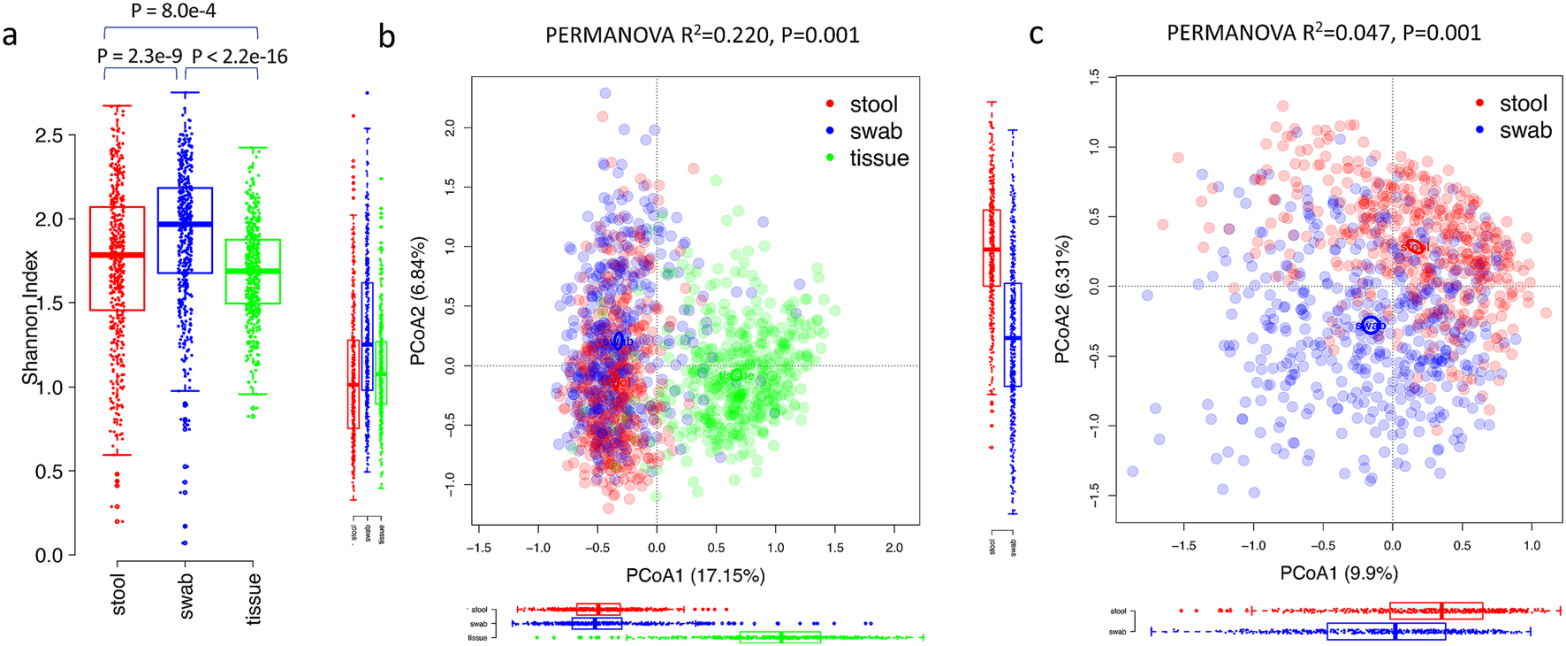
Alpha-diversity and PCoA ordinations of the taxonomic composition of microbial metagenomes at the genus level composition. Color indicates the sample types. **(a)** Alpha diversity across sample types. Differences between sample types were tested with Wilcoxon Rank Sum test. **(b)** Mucosal tissue samples formed a distinct cluster from stool and swab samples. Ellipses indicate 95% confidence limits of the centroids (added with function ‘ordiellipse’ in R package ‘vegan’). **(c)** Separation of stool and swab samples. The boxplots below and on the left of the PCoA plots showed the distribution of coordinates of stool, swab and mucosal tissue samples on PCoA1 and PCoA2. The boxplots showed the median, 25th and 75th percentile.

We characterized the taxonomic composition and functional pathways of 1397 metagenomes and found substantial variation by sample type. Shannon diversity at the genus level was significantly different between sample types, with mucosal tissue samples of the lowest diversity and swab the highest (Fig. 1a). Shannon diversity at the species level and strain level were significantly different between stool and swab and between swab and tissue, but the stool-tissue difference was significant at strain level but not at species level (Fig. S1). PCoA ordinations of genus level composition showed a distinct cluster of mucosal tissue samples (Fig. 1b). A PCoA ordination in which mucosal tissue samples were excluded in order to better visualize the stool and swab samples showed clear separation of their 95% confidence limits as well (Fig. 1c). A PERMANOVA test indicated that the genus level composition was significantly associated with sample type (P = 0.001, with 999 permutations). The differences across stool, swab and mucosal tissue samples explained 22.0% of the variance, while the differences between stool and swab explained 4.7%, further supporting the observation that mucosal tissue samples were more distinct compared to stool and swab. We also tested whether the beta-dispersion were different between sample types. Mucosal tissue samples have significantly lower dispersion than stool and swab (Average distance to centroid within group: stool: 0.273 ± 0.055, swab: 0.281 ± 0.071, mucosal tissue: 0.241 ± 0.070, TukeyHSD.betadisper, P < 0.05), while the dispersion of stool and swab were not significantly different. The differences between mucosal tissue and the other sample types might therefore be caused both by differences in dispersion and differences in the centroid. Microbial taxonomic composition at other levels from phylum to species levels were also significantly associated with sample type (Table S1). The PERMANOVA tests and PCoA ordinations demonstrate that the microbial metagenomes sampled with different methods were different at the community level.

**Figure 2:**
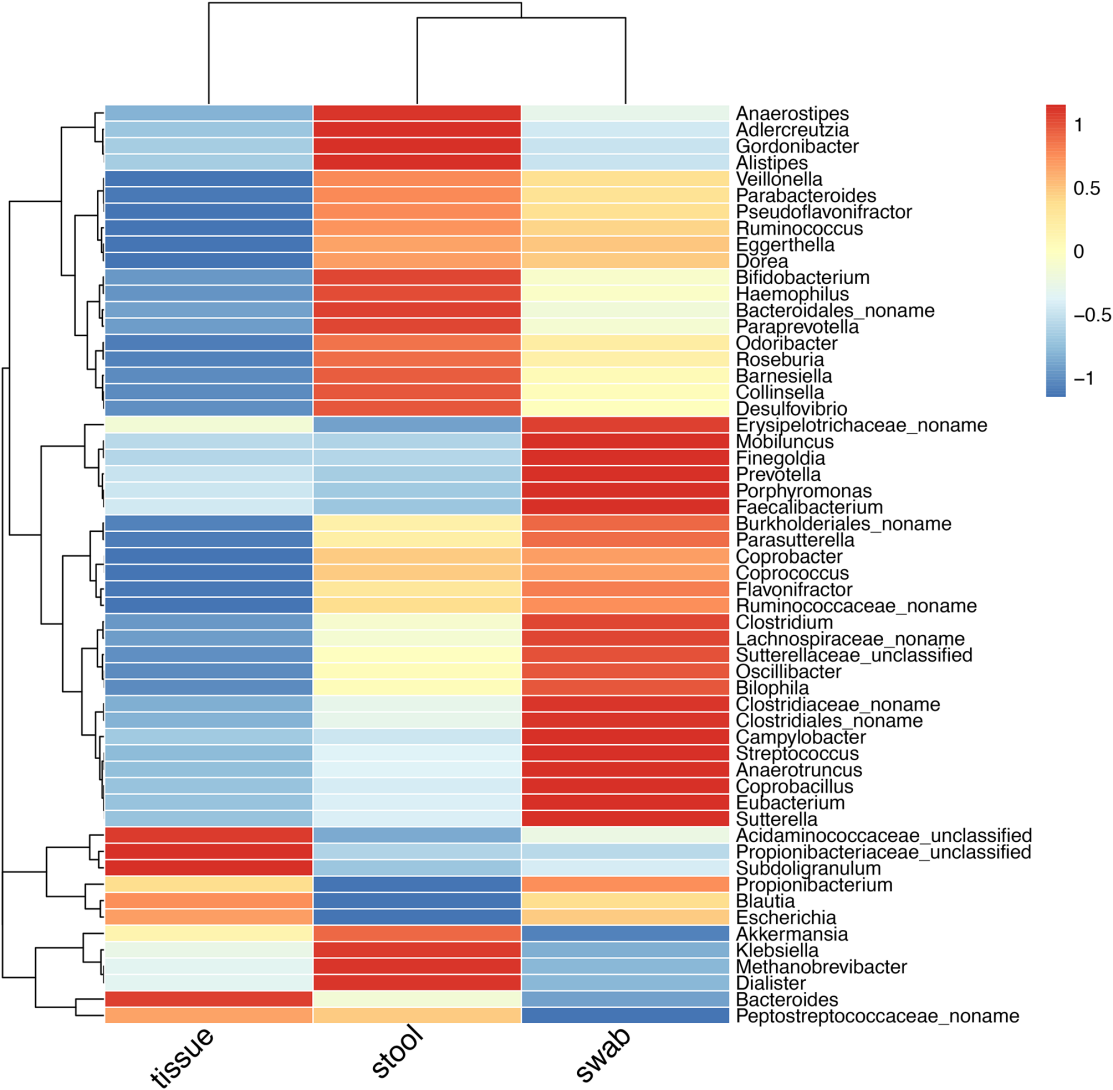
Heatmap of genera that were significantly different between sample types (FDR < 0.05). Keys indicate the z-scores of averaged taxonomic abundance.

In order to identify differentially abundant taxa, we used a linear mixed-effects model to compare the sample types in pairs (Model 1). Among the 60 genera with presence in > 10% samples, 56 were different between at least one pair of sample types, with 35 significantly different between stool and swab samples, 53 between stool and tissue, and 51 between swab and tissue (Fig. 2). Because the sequencing depths were different between sample types (Fig. S2), we also utilized an analysis pipeline based on ALDEx2^18^ to verify the results from the linear regression models. ALDEx2 attempts to explicitly correct for compositional artifacts by transforming the taxonomic composition as the probability of observing the counts and using centered log ratio normalization that is less affected by compositionality. ALDEx2 was used to estimate the difference of genus abundance between pairwise samples. ALDEx2, however, was not designed to adjust for covariates so the ALDEx2 models were run as univariate models. The differential abundance of the 56 taxa across sample types were supported by results from ALDEx2, except for *Paraprevotella* and an unknown genus of the Clostridiaceae family (Table S2). P-values from the two methods were generally consistent (Fig. S3a). Tissue samples had higher relative abundance of *Bacteroides*, *Subdoligranulum, Escherichia*, *Blautia* and unclassified genera of the families *Propionibacteriaceae* and *Acidaminococcaceae*. Compared to stool samples, swab samples were enriched in *Propionibacterium*, *Campylobacter*, *Porphyromonas*, *Prevotella*, *Clostridium*, *Streptococcus* and had lower abundance of *Methanobrevibacter*, *Dialister*, *Adlercreutzia*, *Haemophilus*, *Klebsiella*, *Akkermansia*, *Alistipes* and *Paraprevotella*.

### Functional pathways of metagenomes were associated with sample types

**Figure 3:**
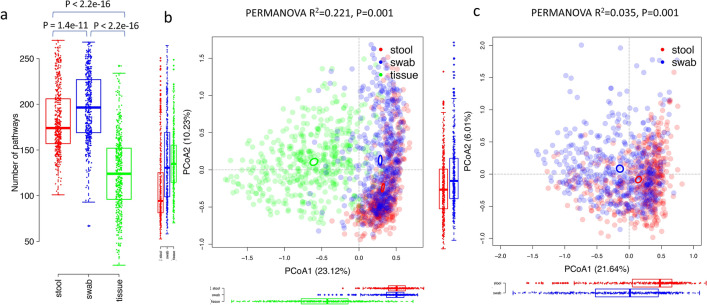
The number of pathways and PCoA ordinations of functional pathways of microbial metagenomes. Color indicates the sample types. **(a)** The number of pathways across samples. Differences between sample types were tested with Wilcoxon Rank Sum test. **(b)** Mucosal tissue samples formed a distinct cluster from stool and swab samples. Ellipses indicate 95% confidence limits of the centroids (added with function ‘ordiellipse’ in R package ‘vegan’). **(c)** Visualization of only stool and swab samples. The boxplots below and on the left of the PCoA plots showed the distribution of coordinates of stool, swab and mucosal tissue samples on PCoA1 and PCoA2. The boxplots showed the median, 25th and 75th percentile.

**Figure 4:**
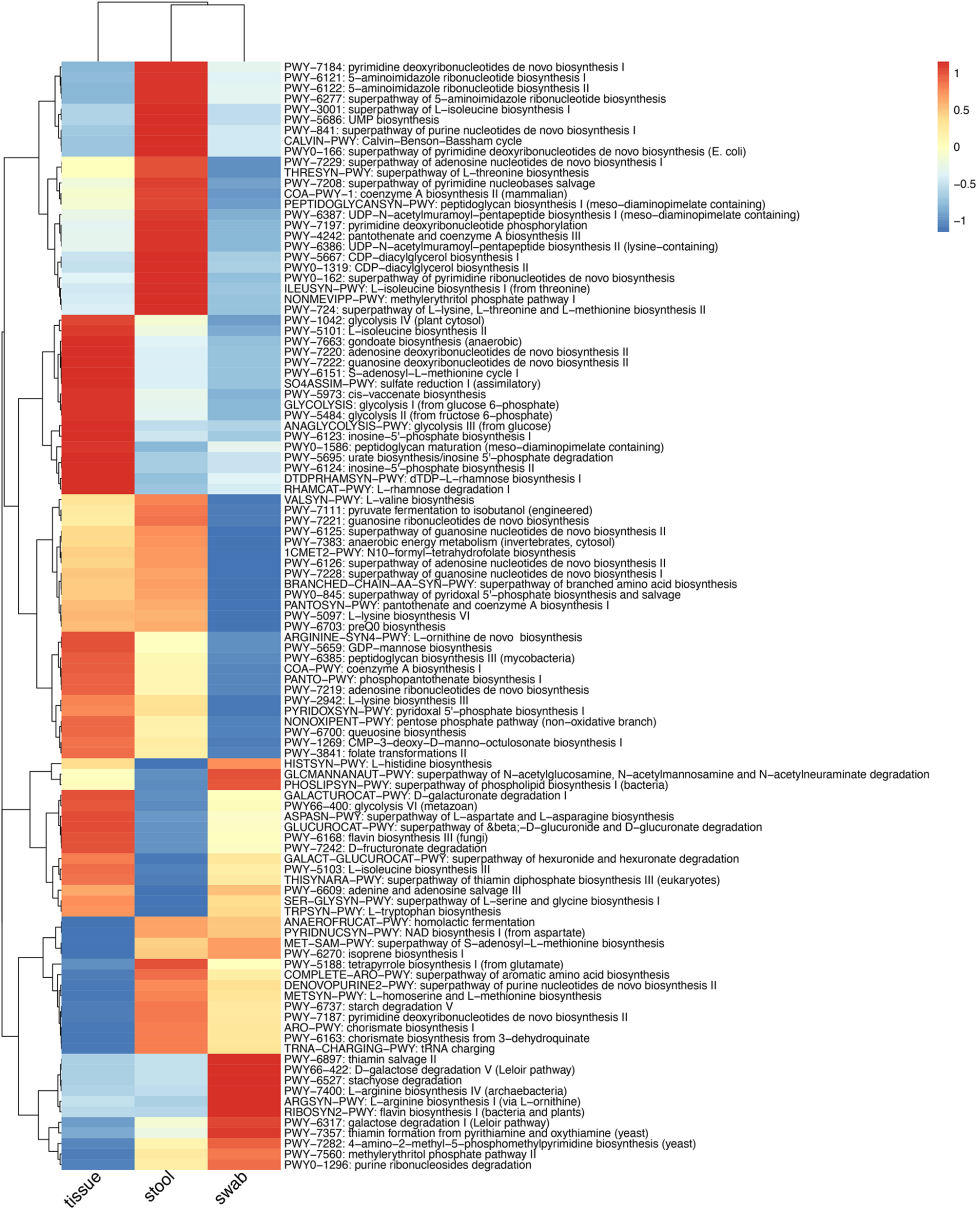
Heatmap of functional pathways that were significantly different between sample types (FDR < 0.05). Keys indicate z-scores of averaged abundance.

The metagenomes of mucosal tissue samples had a higher number of reads that could not be mapped to the UniRef databases after removing host sequences (54% ± 7% compared to 30% ± 10% for stool and 28% ± 9% for swab samples, Wilcoxon rank sum test, P < 2.2E-16), while the number of unmapped reads were not significantly different between stool and swab (Wilcoxon rank sum test, P = 0.997), indicating that the functional genes of mucosal tissue microbiota was less represented in the current database. The numbers of microbial pathways were significantly different between samples types, with the swab samples having the highest number of pathways and mucosal tissues of the lowest number of pathways (Fig. 3a). The PCoA ordinations of functional pathways showed a similar specific cluster of mucosal tissue samples (Fig. 3b), while the stool and swab samples were less separated compared to the PCoA ordination based on genus level composition (Fig. 3c). A PERMANOVA test indicated that functional pathways were also significantly different across sample types (stool, swab and mucosal tissue: R^2^ = 0.221, P = 0.001; stool and swab: R^2^ = 0.035, P = 0.001). The beta-dispersion of sample types were different from each other (Average distance to centroid within group: stool: 0.193 ± 0.043, swab: 0.214 ± 0.051, mucosal tissue: 0.295 ± 0.062, TukeyHSD.betadisper, P < 0.05). We again used a linear mixed effects model to identify the differential functional pathways between samples. In 343 functional pathways with presence in > 10% samples, 318 were significantly different between at least one pair of samples, with 269 of differential abundance for stool-swab comparison, 222 for stool-tissue and 233 for swab-tissue (Fig. 4). Among the 318 significant pathways, only 8 were not supported by the analysis of ALDEx2 (Table S3; Fig. S3b). Stool, swab and mucosal microbiota were enriched for different pathways, reflecting the niche adaption of different microbial communities. Mucosal microbiota was relatively enriched for pathways related to glycolysis and biosynthesis pathways involved in the generation of amino acid l-isoleucine, nucleosides adenosine, guanosine and inosine, and fatty acids gondoate and *cis*-vaccenate (one of the major unsaturated fatty acids, responsible for membrane phospholipid homeostasis in bacteria^19^). The stool and rectal swab microbiomes differed in the pathways related to peptidoglycan, CDP-diacylglycerol, UDP-*N*-acetylmuramoyl-pentapeptide, galactose, stachyose, l-arginine, purine and pyrimidine. Because a large number of functional genes remained unexplored, future expansion of functional databases could provide a better knowledge of the functional differences between these sample types.

### The impact of sample type on the associations between the taxonomic and functional profiles and host factors

**Figure 5:**
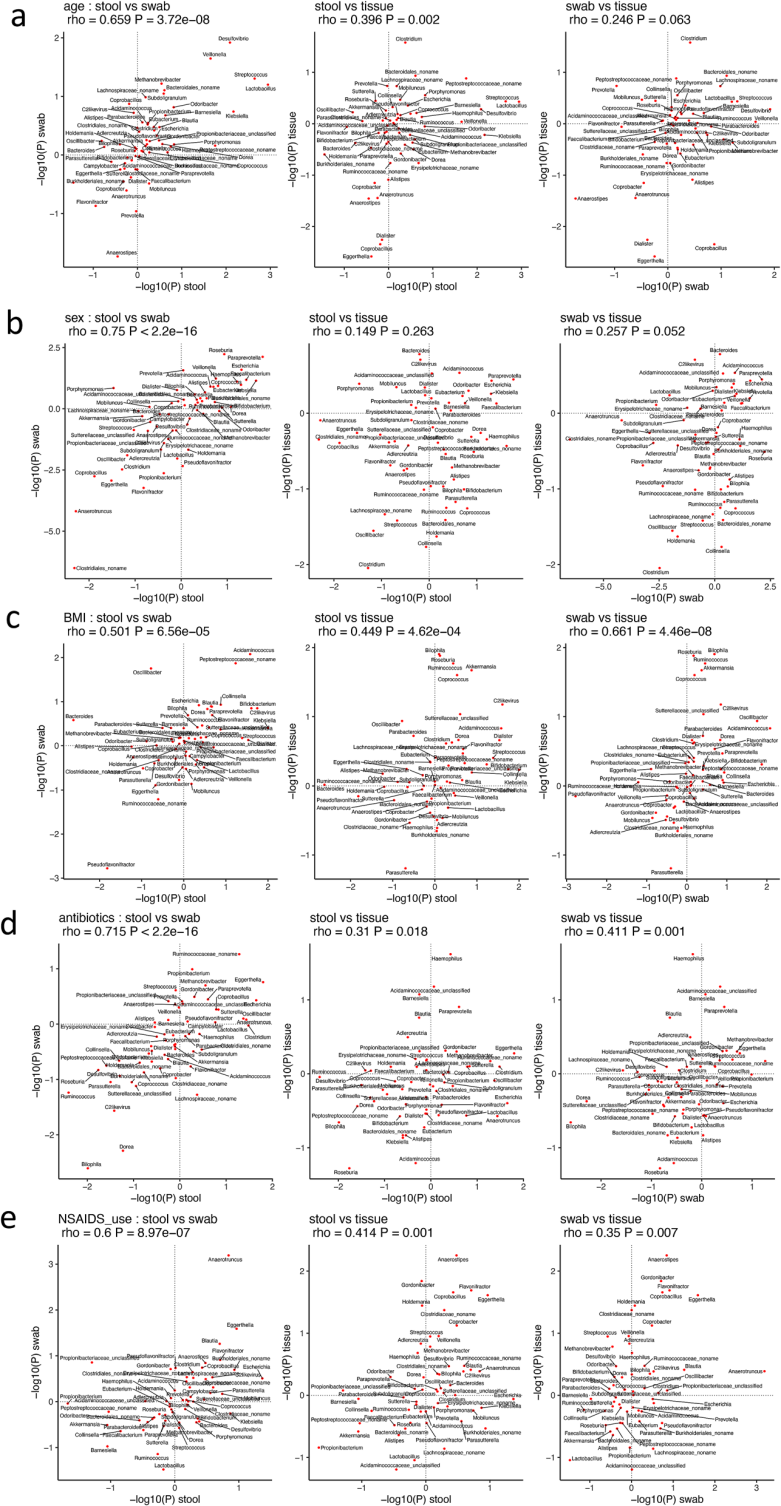
Correlations between the genus level composition inference for age **(a)**, sex **(b)**, BMI **(c)**, antibiotics use **(d)** and NSAIDs use **(e)** between pairwise sample types. The axes showed the p-values that were log10 transformed and multiplied by + 1/−1 to include the direction of changes from the model 2 described in methods.

We built separate mixed effect linear regression models in each sample type to analyze the associations between the normalized abundance of each taxon (details in “Methods”) and each of the host factors age, sex, BMI, antibiotics use and NSAIDs use. To estimate whether the associations inferred in each sample type were consistent, we then tested if the P-values of taxon-factor associations from those models were significantly correlated between each pair of sample types with Spearman’s correlation. The associations between genera and host factors were very highly correlated between stool and swab samples (Fig. 5: left panels) with Spearman’s correlation coefficients of p-value vs. p-value from model 2 (see methods) ranging from 0.501 for BMI to 0.75 for sex. The associations between stool and mucosal tissue samples (Fig. 5: middle panels) were significantly correlated (P < 0.05) except for sex, while the associations between swab and mucosal tissue samples (Fig. 5: right panels) were significantly correlated for BMI, antibiotics use and NSAIDs use but not for age or sex.

**Figure 6:**
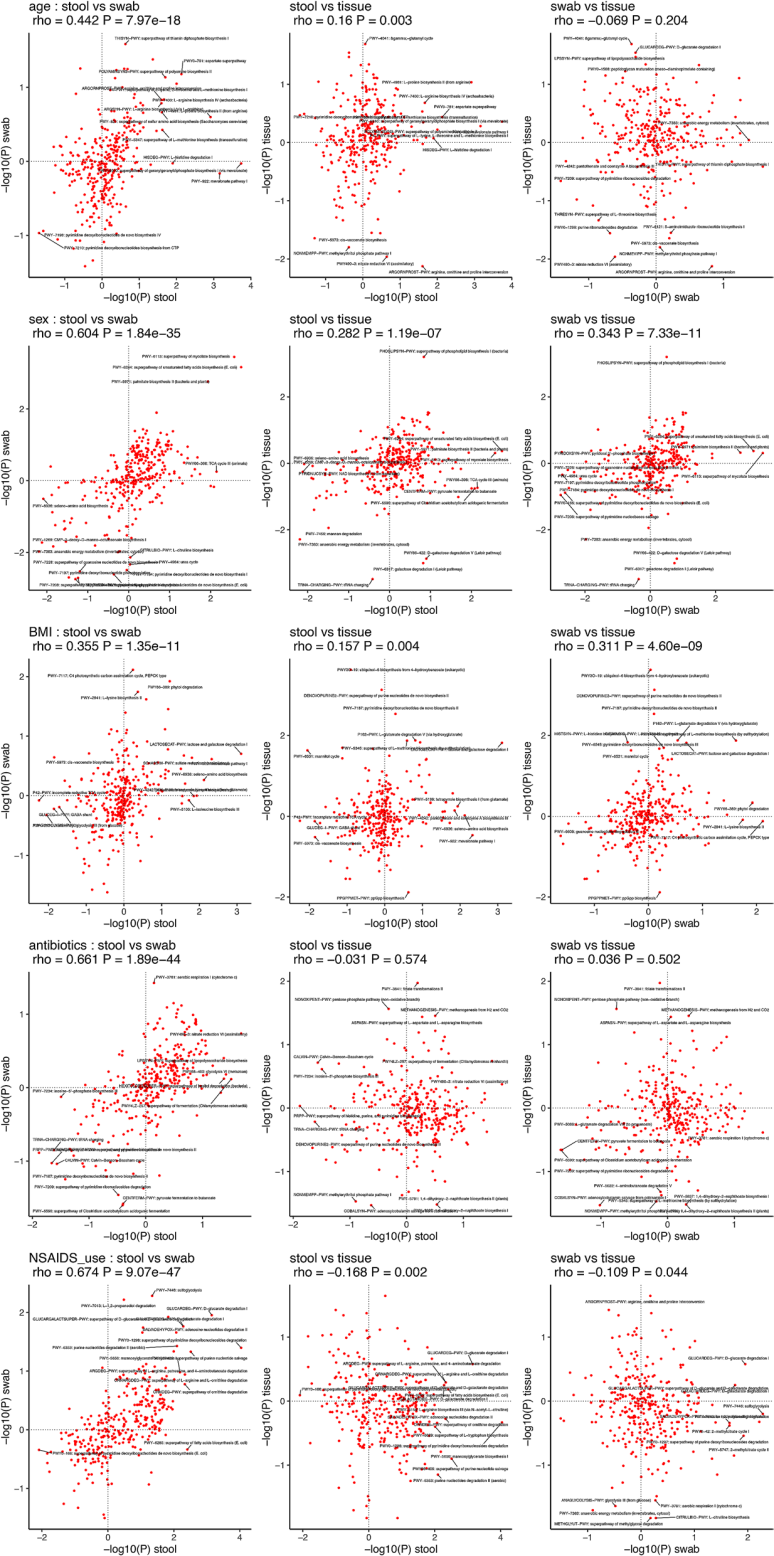
Correlations between the functional pathways inference for age **(a)**, sex **(b)**, BMI **(c)**, antibiotics use **(d)**, NSAIDs use **(e)** and between pairwise sample types. The axes showed the p-values that were log10 transformed and multiplied by + 1/−1 to include the direction of changes from the model 2 described in “Methods”.

The same models were used for analyzing whether the associations between pathways and host factors are consistent across sample types (Fig. 6). As was the case for taxa, the associations between pathways and host factors observed in stool and swab sample types were all highly positively correlated (Fig. 6: left panels). However, comparisons between mucosal tissue and stool (Fig. 6: middle panels) and swab (Fig. 6: right panels) samples showed that the correlations were less consistent, including positive correlation with a smaller coefficient, negative correlation and no correlation. As an alternative visualization, we also generated the correlations of the inferences based on t-values instead of transformed P-values (Fig. S4 and Fig. S5). These observations were generally consistent when using ALDEx2 for statistical modeling instead of the linear models for both taxonomic composition and functional pathways that inference with stool and swab are more consistent than with mucosal tissue (Table S4 and S5). We also reported the results of species level data on their alpha-diversity, beta-diversity (Fig. S6), differential abundance (Fig. S7) and the consistency of their associations with host factors between sample types (Fig. S8), and the findings were generally consistent with those observed at the genus level.

## Discussion

The gut microbiome plays an important role in human health, and a better understanding of the sampling variation of biospecimen types and its influence on the inferred associations between the human gut microbiome and host factors is essential for developing methods utilizing the microbiome. With 1,397 matched stool, rectal swab and mucosal tissue metagenomes for 240 participants, our dataset provided a great opportunity for analyzing the variations of these three matched biospecimens from the same participants. Unsurprisingly, we found that microbial taxonomic composition and functional pathways were different across the three biospecimen types, with the mucosal tissue vs stool and mucosal tissue vs swab differences larger than the difference between stool and swab. However, the inference of host factor and microbiome associations were more consistent between stool and rectal swab than that for mucosal tissue.

The mucosal tissue microbiome had lower alpha diversity and low abundance of most microbes, but was enriched in *Bacteroides, Subdoligranulum, Escherichia* and *Propionibacteriaceae*. *Bacteroides thetaiotaomicron, B. caccae, B. fragilis* and *B. vulgatus* are well known mucin degraders and rely on mucin and other host-derived glycans for colonization^20^. *Propionibacterium* (phylum Actinobacteria) and *Escherichia* (phylum Proteobacteria) were higher in mucosal tissue and swab compared to stool samples, which could be explained by their higher oxygen tolerance. The enrichment of Actinobacteria and Proteobacteria in the mucosa-associated microbiota has been reported in correlation with the intestinal radial colonic oxygen gradient that influences microbiota composition based on their ability to tolerate the oxidative stress^8^. The higher alpha diversity in the rectal swab microbiome compared to the stool and mucosal tissue microbiome is consistent with our previous study^10^ and could be explained by swab sampling from both luminal and mucosal microbes^11^.

Similar to taxonomic composition, the functional pathways in stool and rectal swab samples were less different to each other than mucosal tissue samples. The number of sequencing reads from the mucosal tissue was smaller compared to stool and rectal swab samples due to lower microbial biomass and a higher percentage of human genome DNA contamination (Fig. S2). However, the taxonomic and functional diversity in mucosal tissue microbiome were lower compared to stool and rectal swab samples with all the samples rarefied to the same number of reads, indicating that this difference did not result from the compositional artifacts associated with different sequencing depth we observed between mucosal tissue and stool and swab samples. We also used the compositionally aware pipeline ALDEx2 as an alternative to the linear models analyzing individual taxa and pathways, but no statistical approach can perfectly compensate for the compositionality from sequencing depth variation. ALDEx2 does not allow for inclusion of covariates or adjusting for random effects from the same subject and that might explain the differences between the ALDEx2 and linear models. Future approaches will be needed to explore how much of the differences between mucosal tissues and stool and swab can be explained by these compositional differences.

In order to determine whether the biospecimen type influence the inference of associations between the gut microbiome and host factors, we analyzed microbial associations with age, sex, BMI, antibiotics and NSAIDs use in each of the three sample types. Age, sex, BMI, antibiotic use, and NSAIDs use were pre-selected as adjustment factors in this analysis because of their known relationships with microbiome composition and/or colorectal polyp risk. We found that inferences performed with stool and rectal swab samples were highly correlated with each other for both taxonomic composition and functional pathways, while inference with mucosal tissue was more distinct especially for functional pathways. The relatively poor consistency between the mucosal tissue microbiome and the stool and rectal swab microbiome potentially reflects the niche differences that affect microbial interactions with the environment. It is also possible that the mucus barrier between the mucosal tissue and the lumen makes the mucosal tissue microbiome more sensitive to some host changes that were reflected in the mucosal tissues. For example, a previous study reported that the excessive secretion of mucus glycan could lead to the increase of *Akkermansia* and *Bacteroides* abundance in mucosal tissue but was only extended to stool with an altered mucus barrier^9^. As is the case for comparisons of relative abundance, models of inference are also sensitive to compositional artifacts associated with sequencing depth, although in our study comparisons based on ALDEx2 yielded broadly similar results to comparisons based on compositionally naïve mixed linear models. We were unable to evaluate every factor known to be associated with the gut microbiome, such as transit time^21^. Future studies might wish to consider these factors.

The stool, rectal swab and mucosal tissue biospecimen types examined in this study sample microhabitats in which different microbial communities reside. Stool mostly samples the luminal microbiome, while mucosal tissue biospecimens sample mostly the surface adherent microbiome to the gut epithelium. Rectal swab is considered to sample more mucosal microbiome than stool^8^. Compared to the luminal microbiome, the mucosal microbiome is in close contact to the gut epithelium and may contribute more to nutrient exchange and host immunity induction ^22^. Because of the invasive nature of collecting mucosa specimens, it is of interest how much the mucosal microbiome can be captured by characterizing the stool and rectal swab microbiome. We found that not only are the taxonomic and functional profiles of the mucosa tissue microbiome distinct from that of stool and swab, but that inference of host factor associations were also not consistent, especially for pathways. This indicates that stool and swab are not robust proxies of the mucosa microbiome. Stool samples are widely used for analyzing the gut microbiome, but can be challenging to obtain for some hospitalized patients^12^. We found that the inferences with stool were generally significantly correlated with swab, indicating that stool and swab might generate more comparable inferences than with tissue for some specific host factors.

We note that this study was conducted in individuals with a history of colorectal polyps, so the conclusions may not be generalizable to individuals without a history of polyps. However, all the participants were polyp-free when biospecimens were collected. Our work explicitly compared the microbiota of stool, swab and mucosal tissue, and should provide a useful guide to investigators in the design and interpretation of human studies of the gut microbiota.

## Conclusion

Our study shows that the stool, swab and mucosal tissue microbiota are of different taxonomic and functional profiles, but the stool and swab microbiota are generally less different compared to that of mucosal tissue. When analyzing the associations between microbiota and host factors of age, sex, BMI, antibiotics or NSAIDs use in each sample type, the inference on stool and swab samples were also less different than the inference on mucosal samples. Our study suggests that not only the taxonomic and functional profiles varied by sample types but the inference on their associations with host factors were depending on the sample type as well.

## Methods

### Study population and biospecimen collection

The samples in this study were from the Personalized Prevention of Colorectal Cancer Trial (PPCCT) (https://clinicaltrials.gov/ct2/show/study/NCT01105169, 16/04/2010), but this study is not reporting the results of the clinical trial. All study procedures were performed in accordance with relevant guidelines and regulations as approved by the Vanderbilt Institutional Review Board. Study design and biospecimen collection have been previously described^10^. In brief, participants were randomized to receive for 12 weeks either a personalized dose of placebo (microcrystalline cellulose) or magnesium (magnesium glycinate). Inclusion criteria included aged 40–85, personal history of colorectal polyps, known *TRPM7* rs8042919 genotype, and daily intakes of calcium between 700 and 2000 mg/day and the ratio of calcium to magnesium of 2.6 or greater. Exclusion criteria included pregnancy, breastfeeding, use of medications that may interact with magnesium, or personal history of cancer, colon resection or colectomy, inflammatory bowel disease, organ transplantation, gastric bypass, chronic diarrhea, chronic renal disease, hepatic cirrhosis, chronic ischemic heart disease, or Type I diabetes.

Biospecimens were collected at home or in an in-person study visit at the beginning of the trial (baseline) and at the conclusion of the study 12 weeks later (mean 12.3 ± 1.03 weeks)^10^. Stool samples were collected by study participants at home using a white plastic collection container covering the toilet bowl, aliquoted by the participant into sterile cryovials, and stored in the home freezer until transport with an ice pack to the study visit. Stool was collected up to 3 days prior to the study visit. Rectal swabs and mucosal tissues were collected by the study physician at the study visits. Rectal swabs were collected by inserting a culturette swab through the anal canal, swabbing the distal rectal mucosa, and placing the swab into a cryovial. Rectal mucosal samples were collected through an anoscope using standard mucosal biopsy forceps and these samples were placed into separate storage vials. All three biospecimen types were frozen at − 80 °C until use. During the study, 461 stool samples, 470 swab samples and 466 mucosal tissue samples from 240 participants were collected and included in the analyses. All participants provided at least one biospecimen at baseline or week 12.

### DNA isolation and sequencing

Samples were transferred to a 2 ml tube containing 200 mg of ≤ 106 μm glass beads (Sigma, St. Louis, MO, USA) and 0.3 ml of Qiagen ATL buffer (Qiagen, Valencia, CA, USA), supplemented with lysozyme (20 mg/ml) (Thermo Fisher Scientific, Grand Island, NY, USA). The suspension was incubated at 37 °C for 1 h with occasional agitation. Subsequently the suspension was supplemented with 600 IU of proteinase K and incubated at 60 °C for 1 h. Finally, 0.3 ml of Qiagen AL (Qiagen, Valencia, CA, USA) buffer were added and a final incubation at 70 °C for 10 min was carried out. Bead beating was then performed for 3 min in a Qiagen TissueLyser II (Qiagen, Valencia, CA, USA) at 30 Hz. After a brief centrifugation, supernatants were transferred to a new tube containing 0.3 ml of ethanol. DNA was purified using a standard on-column purification method with Qiagen buffers AW1 and AW2 (Qiagen, Valencia, CA, USA) as washing agents and eluted in 10 mM Tris (pH 8.0).

Whole-genome shotgun metagenomics (WGS) DNA sequencing was performed as previously described ^10^. Briefly, 1 ng of genomic DNA was processed using the Illumina Nextera XT DNA Sample Preparation Kit (Illumina, San Diego, CA, USA). Next, fragmented and tagged DNA was amplified using a limited-cycle PCR program. In this step index 1(i7) and index 2(i5) were added between the downstream bPCR adaptor and the core sequencing library adaptor, as well primer sequences required for cluster formation. The DNA library was purified using Agencourt^®^ AMPure^®^ XP Reagent (Beckman Coulter, Brea, CA). Each sample was quantified and normalized prior to pooling. The DNA library pool was loaded on the Illumina platform reagent cartridge and on the Illumina HiSeq instrument (Illumina, San Diego, CA, USA). For validation of the DNA isolation process, a blank composed of only DNA isolation reagents was included in the DNA extraction process and again in the library preparation. In addition to the isolation blank, the library preparation also included a known bacterial community, ZymoBIOMICS Microbial Community DNA Standard (Zymo Research Corporation, Irvine, CA, USA, Cat#D6305), and a library blank composed of library preparation reagents alone.

### Bioinformatics and statistical analyses

Sequencing output from the Illumina HiSeq4000 platform was converted to fastq format and demultiplexed using Illumina Bcl2Fastq 2.18.0.12. Quality control of the demultiplexed sequencing reads was verified by FastQC. Human genome contamination was removed from the shotgun metagenome sequencing reads with KneadData. The number of reads before and after removing human genome contamination is shown in Fig. S2. The taxonomic composition of the filtered reads was characterized with MetaPhlAn2^23^ while the functional pathways were annotated with HUMAnN2 against the UniRef database^24^. The count tables of taxonomic and functional profiles were rarefied to the minimum number of reads per sample for Shannon diversity, the number of pathways, PCoA, PERMANOVA and beta dispersion calculation to minimize the impact of sequencing depth on multivariate analyses^25^. Because rarefaction decreases the sensitivity of differential abundance analysis^25^, the abundance of taxonomic and functional profiles were normalized as previously described for mixed effects linear models^10^. Unmapped reads were not included in the statistical analyses. Unclassified taxa by MetaPhlAn2 were grouped together. PCoA ordination was generated with Bray–Curtis dissimilarity based on genus level composition and functional pathway abundance respectively with function ‘capscale’ in the R package ‘vegan’. The PERMANOVA test was performed with the function ‘adonis’ in the same package. For each individual genus or pathway, we built linear mixed effects models using the function ‘lme’ in R package ‘nlme’ with method “REML” and random intercepts. The genera and pathways with presence < 10% in all samples were excluded to avoid spurious results based on previous studies^26,27^. P-values were adjusted with the Benjamini–Hochberg method for multiple testing.

Model 1 was used to test the associations between the normalized abundance of genus level composition and functional pathways and biospecimen types (stool, swab or mucosal tissue). Model 1 was performed for each pair of sample types to get the direction of changes and adjusted for host factors.

1 $$ {\text{Genus/pathway  =  sample\_type + treatment*time\_point + antibiotics use  + age + sex + BMI + NSAIDs use + (1/participant)}}  $$

In this model, sample type, treatment, time point, age, sex, BMI, antibiotics and NSAIDs use were fixed effects while participant ID was a random effect. Using pairwise models allowed for direct comparison between sample types. The significance was determined as < 10% FDRs corrected with Benjamini–Hochberg method. Significant genera and pathways identified in this model were plotted as heatmaps with the function ‘pheatmap’.

Model 2 was used to test the associations between the normalized abundance of genus level composition and functional pathways and host factors in each sample type individually.

2 $$ {\text{Genus/pathway  =  treatment*time\_point + antibiotics use  + age + sex + BMI + NSAIDs use  + (1/participant)}} $$

In this model, treatment, time point, age, sex, BMI, antibiotics and NSAIDs use were fixed effects while participant ID is a random effect. To estimate whether the associations with each host factor inferred from Model 2 were consistent across three sample types, we tested the correlations of the transformed P-values in each pair of sample types with Spearman’s correlation. The P-values were log transformed and multiplied by 1 or −1 to include the direction of changes. For example, the transformed P-values of the associations between each genus and age in stool were analyzed and plotted against the transformed P-values of the association in swab. The scatter plots were generated with ‘ggplot2’. The correlation statistics and plots of t-values from the model 2 are shown in Fig. S4 and S5.

Because of the compositional nature of the shotgun metagenome sequencing data and the variation of sequencing depth across samples, we also utilized ALDEx2^28^ to confirm that the results we observed with the mixed effects linear regression models were not due to compositional artifact of the sequencing data. ALDEx2 models the count data as the probability of observing the counts and transforms the data with a log ratio geometric mean based normalization to minimize compositional artifacts. ALDEx2 was run on each pair of samples separately. Because ALDEx2 does not support models adjusted for covariates, the associations were tested with one variable models. The counts tables were used as inputs to generate 128 Dirichlet Monte-Carlo instances using module ‘aldex.clr’, and then inferences were generated with the instances using module ‘aldex.ttest’ following the developer’s instructions.

### Ethics approval and consent to participate

All study procedures were approved by the Vanderbilt Institutional Review Board. All participants in this study gave written informed consent.

## Supplementary Information

Supplementary Information.

Supplementary Table S2.

Supplementary Table S3.

## Data Availability

The metagenomes sequences analyzed in this study are available at NCBI with accession ID PRJNA693850. Scripts used in this study are available at https://github.com/ssun6/StoolSwabTissue.

## References

[1] Arthur JC (2014). Microbial genomic analysis reveals the essential role of inflammation in bacteria-induced colorectal cancer. Nat. Commun..

[2] Kostic AD, Xavier RJ, Gevers D (2014). The microbiome in inflammatory bowel disease: Current status and the future ahead. Gastroenterology.

[3] Graham C, Mullen A, Whelan K (2015). Obesity and the gastrointestinal microbiota: A review of associations and mechanisms. Nutr. Rev..

[4] Qin J (2012). A metagenome-wide association study of gut microbiota in type 2 diabetes. Nature.

[5] Donaldson GP, Lee SM, Mazmanian SK (2016). Gut biogeography of the bacterial microbiota. Nat. Rev. Microbiol..

[6] Espey MG (2013). Role of oxygen gradients in shaping redox relationships between the human intestine and its microbiota. Free Radic. Biol. Med..

[7] Friedman, E. S. *et al.* Microbes vs. chemistry in the origin of the anaerobic gut lumen. *Proc. Natl. Acad. Sci.* **115**, 4170–4175 (2018). 10.1073/pnas.1718635115 PMC5910840 29610310

[8] Albenberg, L. *et al.* Correlation between intraluminal oxygen gradient and radial partitioning of intestinal microbiota. *Gastroenterology* **147**, 1055–1063.e1058 (2014). 10.1053/j.gastro.2014.07.020 PMC4252572 25046162

[9] Glymenaki M (2017). Compositional changes in the gut mucus microbiota precede the onset of colitis-induced inflammation. Inflamm. Bowel Dis..

[10] Jones RB (2018). Inter-niche and inter-individual variation in gut microbial community assessment using stool, rectal swab, and mucosal samples. Sci. Rep..

[11] Choudhury R, Kleerebezem M, Middelkoop A, Bolhuis JE (2019). Legitimate and reliable determination of the age-related intestinal microbiome in young piglets; rectal swabs and fecal samples provide comparable insights. Front. Microbiol..

[12] Bassis CM (2017). Comparison of stool versus rectal swab samples and storage conditions on bacterial community profiles. BMC Microbiol..

[13] Fair K (2019). Rectal swabs from critically ill patients provide discordant representations of the gut microbiome compared to stool samples. Msphere.

[14] Vaga S (2020). Compositional and functional differences of the mucosal microbiota along the intestine of healthy individuals. Sci. Rep..

[15] Mas-Lloret J (2020). Gut microbiome diversity detected by high-coverage 16S and shotgun sequencing of paired stool and colon sample. Sci. Data.

[16] Brooks JP (2015). The truth about metagenomics: Quantifying and counteracting bias in 16S rRNA studies. BMC Microbiol..

[17] Ahn J-H, Kim B-Y, Song J, Weon H-Y (2012). Effects of PCR cycle number and DNA polymerase type on the 16S rRNA gene pyrosequencing analysis of bacterial communities. J. Microbiol..

[18] Fernandes AD (2014). Unifying the analysis of high-throughput sequencing datasets: Characterizing RNA-seq, 16S rRNA gene sequencing and selective growth experiments by compositional data analysis. Microbiome.

[19] Zhang Y-M, Rock CO (2008). Membrane lipid homeostasis in bacteria. Nat. Rev. Microbiol..

[20] Martens EC, Chiang HC, Gordon JI (2008). Mucosal glycan foraging enhances fitness and transmission of a saccharolytic human gut bacterial symbiont. Cell Host Microbe.

[21] Falony G (2016). Population-level analysis of gut microbiome variation. Science.

[22] Eckburg, P. B. *et al.* Diversity of the human intestinal microbial flora. *Science* **308**, 1635–1638 (2005). 10.1126/science.1110591 PMC1395357 15831718

[23] Truong DT (2015). MetaPhlAn2 for enhanced metagenomic taxonomic profiling. Nat. Methods.

[24] Franzosa EA (2018). Species-level functional profiling of metagenomes and metatranscriptomes. Nat. Methods.

[25] Weiss S (2017). Normalization and microbial differential abundance strategies depend upon data characteristics. Microbiome.

[26] Scepanovic P (2019). A comprehensive assessment of demographic, environmental, and host genetic associations with gut microbiome diversity in healthy individuals. Microbiome.

[27] Hill CJ (2017). Evolution of gut microbiota composition from birth to 24 weeks in the INFANTMET Cohort. Microbiome.

[28] Fernandes, A. D., Macklaim, J. M., Linn, T. G., Reid, G. & Gloor, G. B. ANOVA-like differential expression (ALDEx) analysis for mixed population RNA-Seq. *PLoS One* **8**, e67019 (2013). 10.1371/journal.pone.0067019 PMC3699591 23843979

