# Supplementary material for: On the robustness of inference of association with the gut microbiota in stool, rectal swab and mucosal tissue samples
Source: Sci Rep. 2021 Jul 21;11:14828. doi: 10.1038/s41598-021-94205-5 (PMC8295290; doi:10.1038/s41598-021-94205-5)
Supplement: Supplementary file 1 — Supplementary Information. [file 41598_2021_94205_MOESM1_ESM.pdf]

On the robustness of inference of association with the gut microbiota in stool, rectal swab and mucosal tissue samples

Shan Sun[1], Xiangzhu Zhu[2], Xiang Huang[2], Harvey J. Murff[2], Reid M. Ness[2], Douglas L. Seidner[3], Alicia A. Sorgen[1], Ivory C. Blakley[1], Chang Yu[4], Qi Dai[2], M. Andrea Azcarate-Peril[5], Martha J. Shrubsole[2*], Anthony A. Fodor[1*]

[1] Department of Bioinformatics and Genomics, University of North Carolina at Charlotte, Charlotte, NC, USA.

[2] Department of Medicine, Vanderbilt University Medical Center, Nashville, TN, USA.

[3] Digestive Disease and Surgical Institute, Cleveland Clinic, Cleveland, OH, USA.

[4] Department of Biostatistics, Vanderbilt University Medical Center, Nashville, TN, USA.

[5] Department of Medicine and Microbiome Core Facility, School of Medicine, University of North Carolina, Chapel Hill, NC, USA.

Corresponding Author:

Anthony A. Fodor

Department of Bioinformatics and Genomics

University of North Carolina at Charlotte

9331 Robert D. Snyder Rd, Room 361

Charlotte, NC 28223

afodor@uncc.edu

Supplementary Table

Table S1. PERMANOVA test of the association between sample types and taxonomic composition of microbial metagenomes from phylum to species level.

	Stool, swab and tissue		Stool and swab	
	R^2	P	R^2	P
phylum	0.187	0.001	0.039	0.001
class	0.252	0.001	0.039	0.001
order	0.249	0.001	0.050	0.001
family	0.232	0.001	0.045	0.001
genus	0.220	0.001	0.048	0.001
species	0.170	0.001	0.035	0.001

Table S4. Correlation of the associations between taxonomic composition and host factors in stool, swab and tissue samples estimated with ALDEx2.

	**stool vs swab**		**stool vs tissue**		**swab vs tissue**	
	rho	P	rho	P	rho	P
age	0.193	0.025	-0.037	0.731	-0.083	0.430
BMI	-0.180	0.038	0.020	0.849	0.006	0.955
sex	0.427	3.55E-07	0.001	0.991	0.037	0.726
NSAIDS_use	0.206	0.017	0.022	0.840	0.045	0.668
antibiotics	0.326	1.70E-04	-0.070	0.515	-0.174	0.099

Table S5. Correlation of the associations between functional pathways and host factors in stool, swab and tissue samples estimated with ALDEx2.

	**stool vs swab**		**stool vs tissue**		**swab vs tissue**	
	rho	P	rho	P	rho	P
age	0.105	0.026	-0.112	0.031	-0.012	0.814
BMI	-0.028	0.549	0.138	0.008	-0.025	0.626
sex	0.376	2.22E-16	0.008	0.883	-0.033	0.522
NSAIDS_use	0.41	2.18E-19	0.15	0.004	-0.028	0.589
antibiotics	0.308	3.63E-11	0.062	0.232	0.117	0.024

Table S6. PERMANOVA test of the association between host factors and genus level composition of microbial metagenomes.

	**stool**		**swab**		**tissue**	
	R^2	P	R^2	P	R^2	P
age	0.0063	0.001	0.0045	0.008	0.0038	0.05
BMI	0.0079	0.001	0.0041	0.023	0.0042	0.041

sex	0.0134	0.001	0.0168	0.001	0.0038	0.069
NSAIDS_use	0.0031	0.137	0.0046	0.012	0.0018	0.607
antibiotics	0.0118	0.001	0.0065	0.002	0.0049	0.041

Table S7. PERMANOVA test of the association between host factors and pathway abundance of microbial metagenomes.

	**stool**		**swab**		**tissue**	
	R^2	P	R^2	P	R^2	P
age	0.0062	0.003	0.0023	0.279	0.0019	0.533
BMI	0.0028	0.167	0.0020	0.456	0.0026	0.18
sex	0.0056	0.008	0.0036	0.078	0.0049	0.013
NSAIDS_use	0.0061	0.008	0.0029	0.143	0.0019	0.532
antibiotics	0.0040	0.093	0.0028	0.272	0.0028	0.308

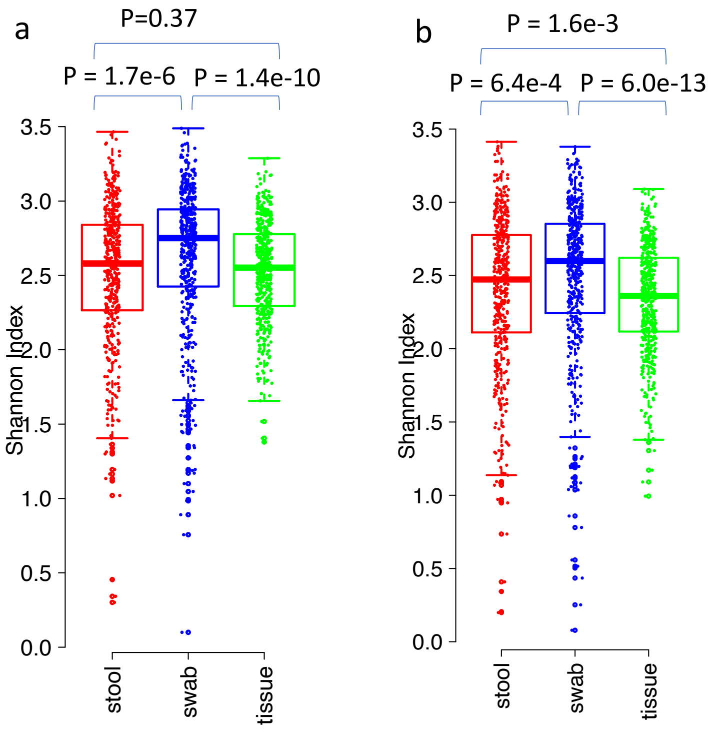

Fig. S1. Alpha-diversity of the taxonomic composition of microbial metagenomes at the species (a) and strain (b) level. The count tables were rarefied to the minimum number of reads per sample to correct for the differences in sequencing depth. Color indicates the sample types. Differences between sample types were tested with Wilcoxon Rank Sum test.

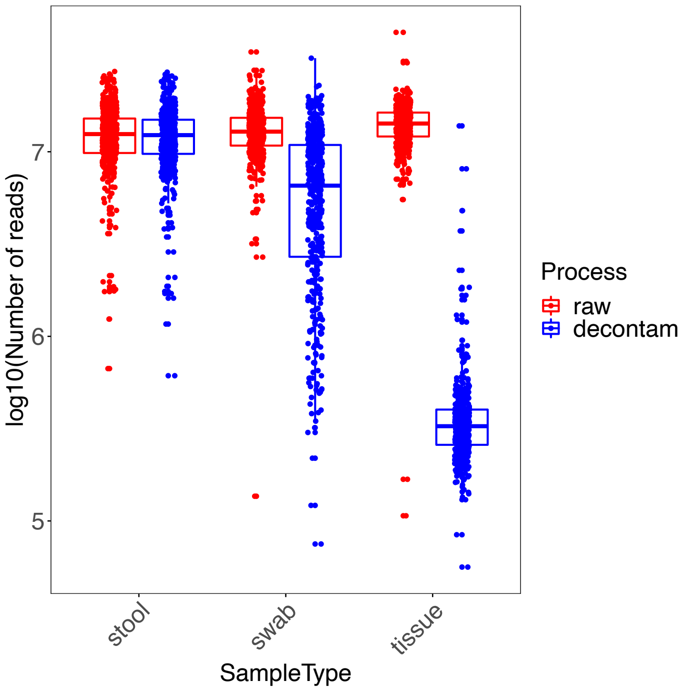

Fig. S2. Number of sequencing reads before and after removing human genome contamination in each sample type.

a

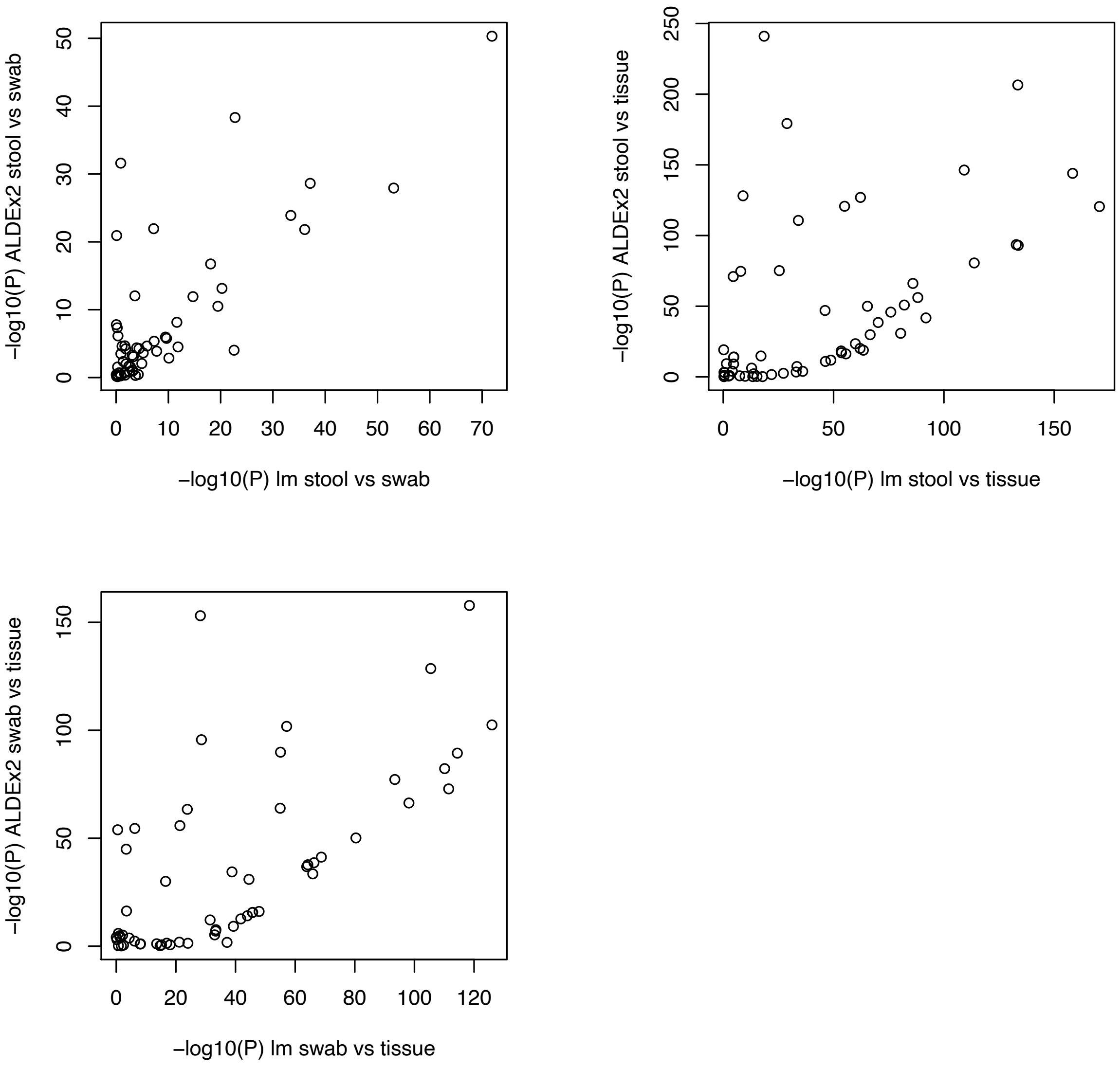

−log10(P) ALDEx2 stool vs swab
−log10(P) lm stool vs swab
−log10(P) ALDEx2 stool vs tissue
−log10(P) lm stool vs tissue
−log10(P) ALDEx2 swab vs tissue
−log10(P) lm swab vs tissue

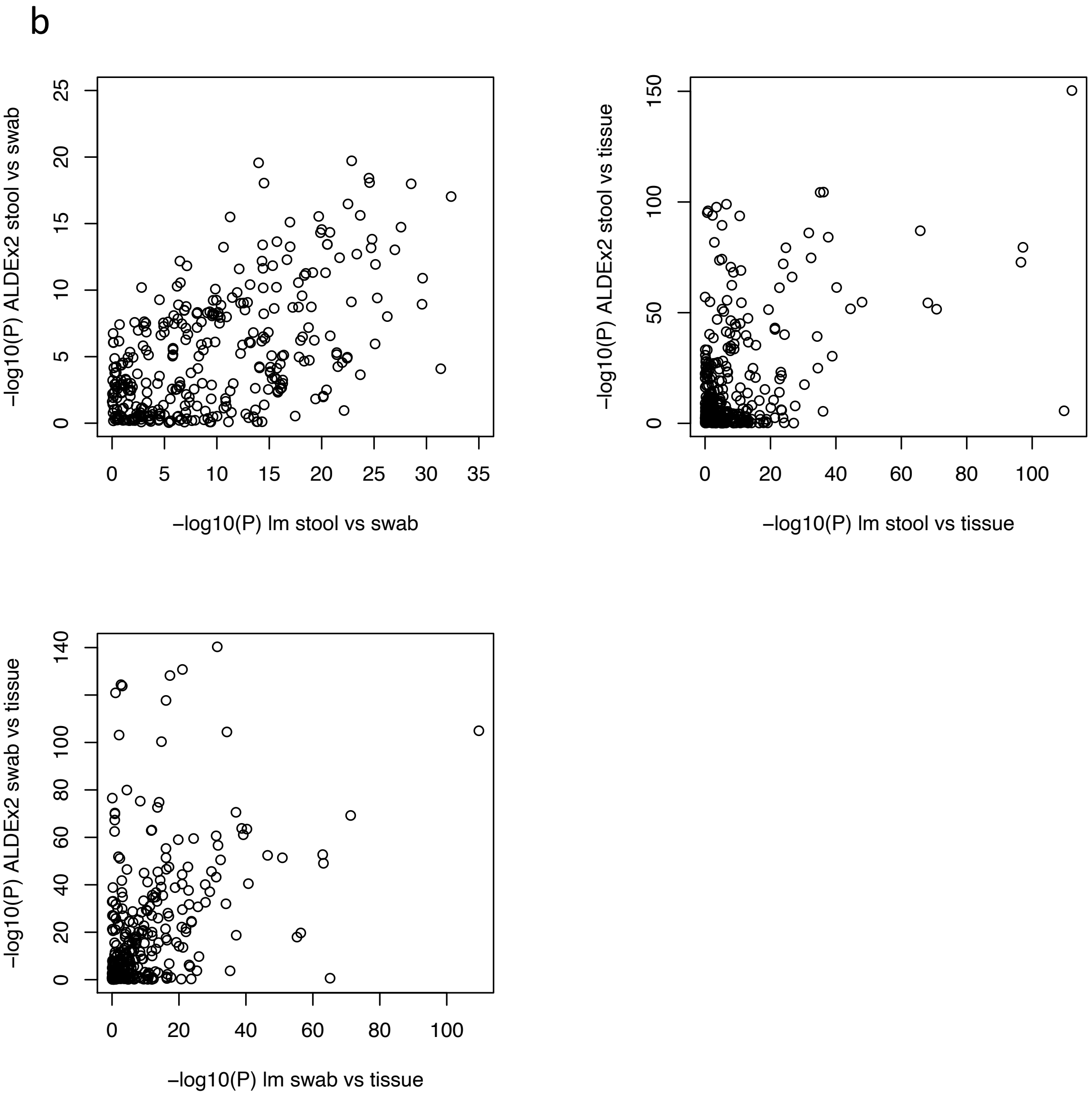

Fig. S3. Comparison of P values from ALDEx2 and mixed effects linear models for each pair of sample types for genus (a) and pathway abundance (b).

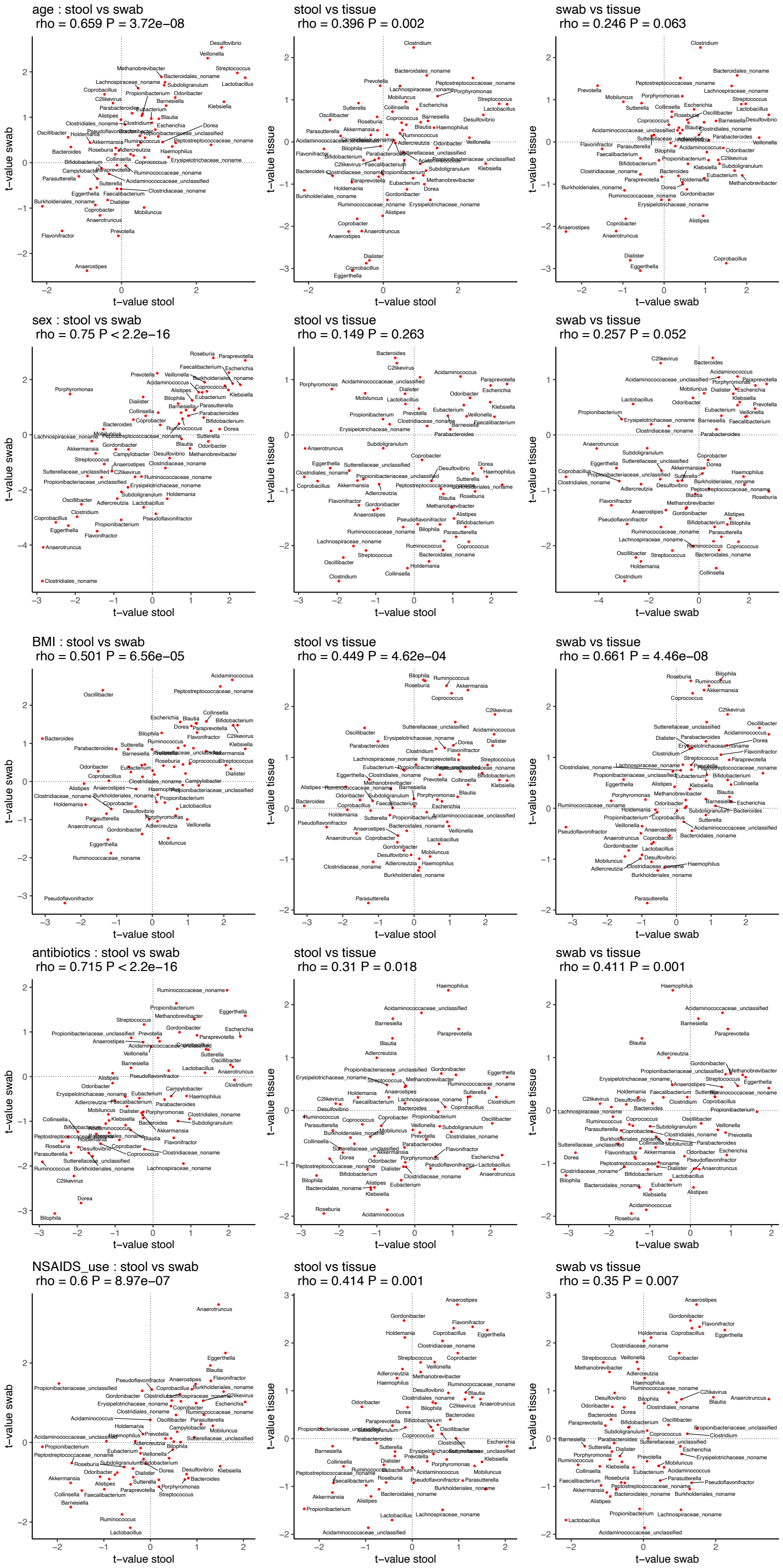

Fig. S4. Correlations between the genus level composition inference (model 2 t-values) for age (a), sex (b), BMI (c), antibiotics use (d) and NSAIDs use (e) between pairwise sample types. The axes were the t-values from the model2 described in methods

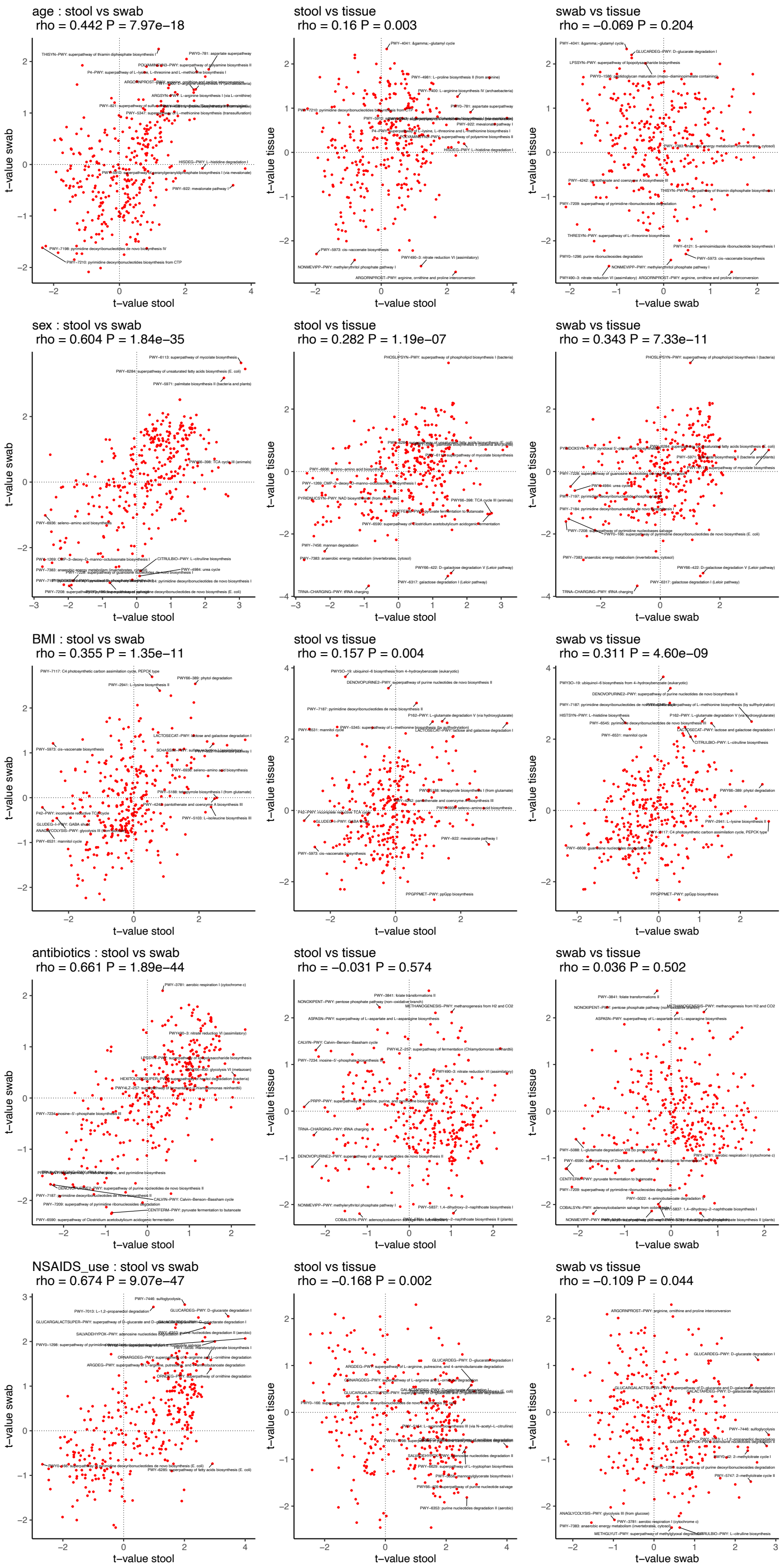

Fig. S5. Correlations between the pathway abundance inference (model 2 t-values) for age (a), sex (b), BMI (c), antibiotics use (d) and NSAIDs use (e) between pairwise sample types. The axes were the t-values from the model2 described in methods.

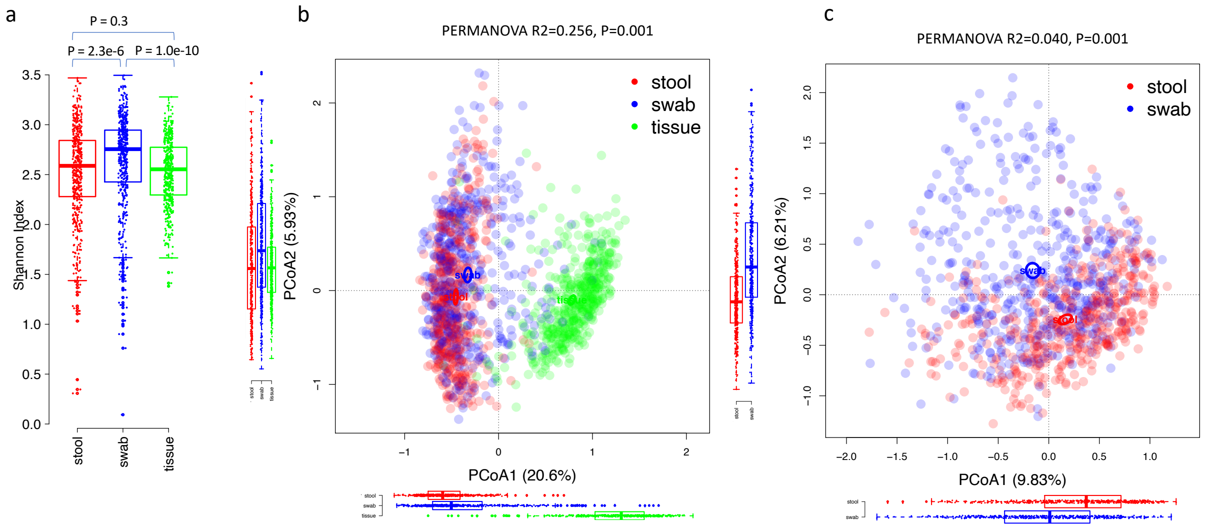

Fig. S6. Alpha-diversity and PCoA ordinations of the taxonomic composition of microbial metagenomes at the **species** level composition. Color indicates the sample types. (a) Alpha diversity across sample types. Differences between sample types were tested with Wilcoxon Rank Sum test. (b) Mucosal tissue samples formed a distinct cluster from stool and swab samples. Ellipses indicate 95% confidence limits of the centroids (added with function 'ordiellipse' in R package 'vegan') (c) Separation of stool and swab samples. The boxplots below and on the left of the PCoA plots showed the distribution of coordinates of stool, swab and mucosal tissue samples on PCoA1 and PCoA2. The boxplots showed the median, 25th and 75th percentile.

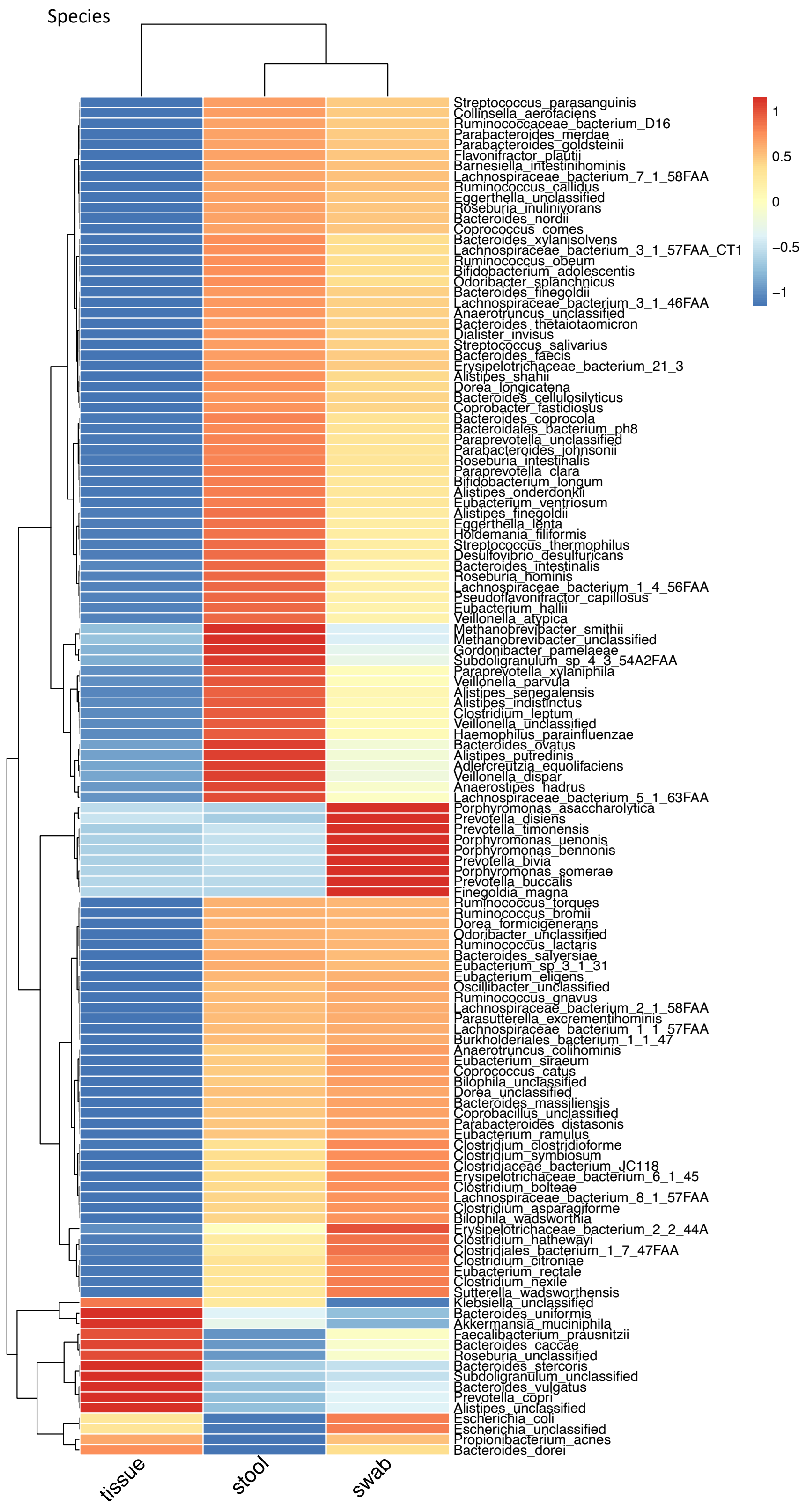

Fig. S7. Heatmap of **species** that were significantly different between sample types (FDR<0.05) with a prevalence > 10%. Keys indicate the z-scores of averaged taxonomic abundance.

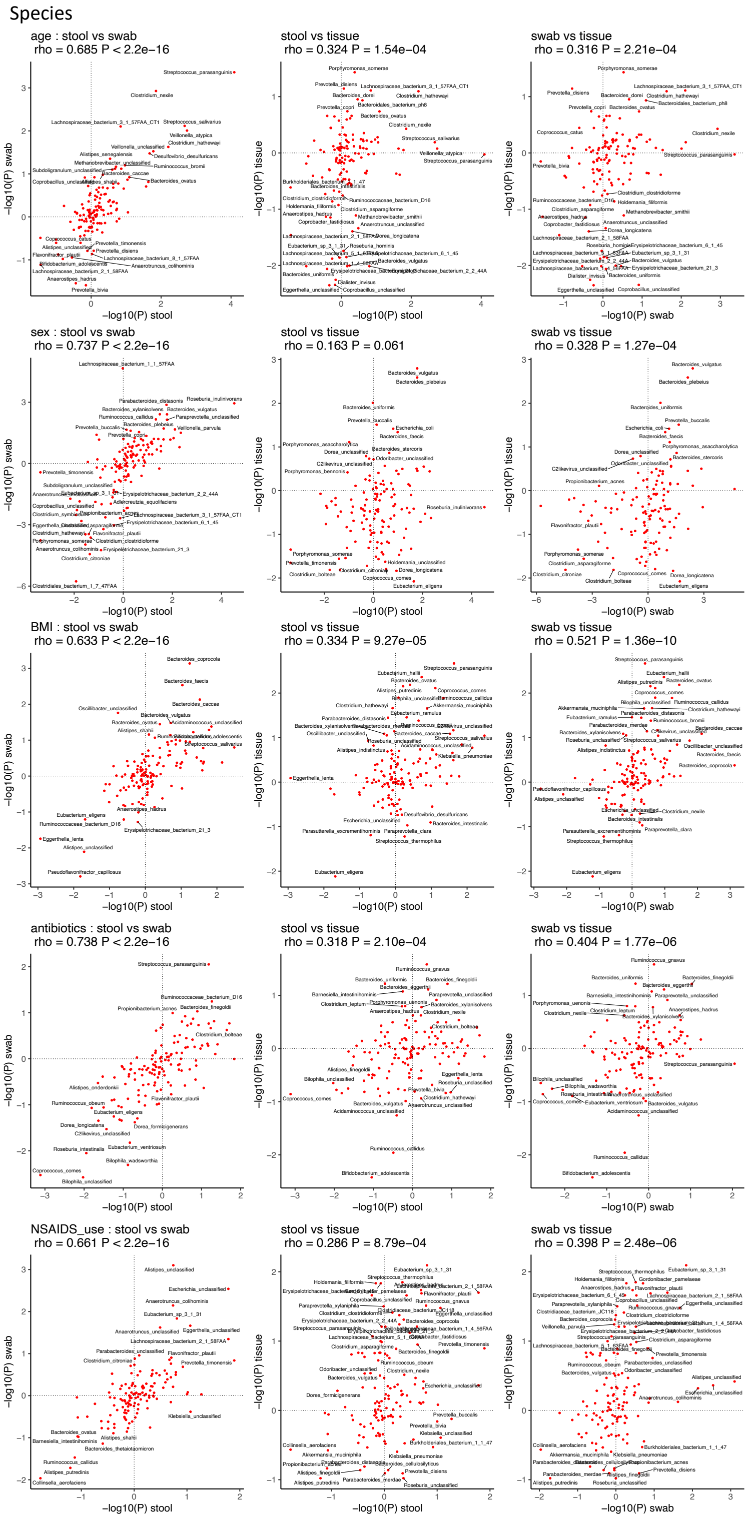

Fig. S8. Correlations between the **species** level composition inference for age (a), sex (b), BMI (c), antibiotics use (d) and NSAIDs use (e) between pairwise sample types. The axes showed the p-values that were log10 transformed and multiplied by +1/-1 to include the direction of changes from the model 2 described in methods.
